# A Novel Anti-HER2 Bispecific Antibody With Potent Tumor Inhibitory Effects *In Vitro* and *In Vivo*

**DOI:** 10.3389/fimmu.2020.600883

**Published:** 2021-02-17

**Authors:** Mehdi Mohammadi, Mahmood Jeddi-Tehrani, Forough Golsaz-Shirazi, Mohammad Arjmand, Tannaz Bahadori, Mohammad Ali Judaki, Fariba Shiravi, Hengameh Ahmadi Zare, Farzaneh Notash Haghighat, Maryam Mobini, Mohammad Mehdi Amiri, Fazel Shokri

**Affiliations:** ^1^ Department of Immunology, School of Public Health, Tehran University of Medical Sciences, Tehran, Iran; ^2^ Department of Biochemistry, Pasteur Institute of Iran, Tehran, Iran; ^3^ Monoclonal Antibody Research Center, Avicenna Research Institute, Academic Center for Education, Culture and Research (ACECR), Tehran, Iran

**Keywords:** HER2, DVD-Ig, monoclonal antibody, bispecific antibody, cancer immunotherapy

## Abstract

Overexpression of HER2 has been reported in many types of cancer, making it a perfect candidate for targeted immunotherapy. The combination of two FDA approved monoclonal antibodies (mAbs), trastuzumab and pertuzumab, has more robust anti-tumor activity in patients with HER2-overexpressing breast cancer. We recently produced a new humanized anti-HER2 mAb, hersintuzumab, which recognizes a different epitope than trastuzumab and pertuzumab on HER2. This mAb, in combination with trastuzumab, exhibits more potent anti-tumor activity than each parental mAb alone. Here we have developed a novel bispecific anti-HER2 antibody (BsAb) designated as trasintuzumab, composed of trastuzumab and hersintuzumab, using dual variable domain immunoglobulin (DVD-Ig) technology. Both variable domains of trasintuzumab are fully functional and have similar affinities to the parental mAbs and are also able to bind to natural HER2 on the surface of several HER2-expressing cell lines. Trasintuzumab was found to inhibit the growth of different types of tumor cell lines through suppression of the AKT and ERK signaling pathways as efficiently as the combination of the parental mAbs. It also induced tumor regression as potently as the combination of the two mAbs in nude mice bearing ovarian and gastric cancer xenografts. Our data suggest that trasintuzumab may be a promising BsAb therapeutic candidate for the treatment of HER2-overexpressing cancers.

## Introduction

The human HER (human epidermal growth factor receptor) family includes HER1 (ErbB1, also known as EGFR), HER2 (ErbB2, c-erbB2, or HER2/neu), HER3 (ErbB3), and HER4 (ErbB4) molecules. These receptors are structurally similar and their hetero/homo dimerizations play important roles in cell differentiation, growth, and survival (1). Among this family, HER2 is studied more extensively. It is a 185 kDa protein with four extracellular domains (ECD) and an intracellular tyrosine kinase domain, which mainly signals through phosphatidylinositol-3 kinase (PI3K) and mitogen-activated protein kinase (MAPK) signaling pathways (2). There is no identified ligand for HER2 and it forms heterodimers with other members of the HER family to activate downstream signaling pathways. Overexpression of HER2 is associated with disease prognosis and has been observed in approximately 25%–30% of breast cancers, 6%–35% of gastric cancers, 9%–32% of ovarian cancers, and 25%–78% of prostate cancers (3).

Since the discovery of the HER2 role in tumor progression, extensive efforts have been made to produce monoclonal antibodies (mAbs) against this target. Trastuzumab is the first mAb against domain IV of HER2 (4), which was approved by the FDA for breast cancer in 1998 and for metastatic stomach cancer in 2010. Pertuzumab is another mAb that targets domain II of HER2 (5). Since trastuzumab and pertuzumab bind to different domains of HER2 and are non-competitive, their combination therapy showed synergistic effects (6) and was later approved for treatment of metastatic breast cancer in 2012. Based on the synergistic effects of trastuzumab and pertuzumab against different epitopes of HER2, efforts have been made globally to develop novel antibodies for combination therapies. Bong-Kook and colleagues developed a humanized anti HER2 mAb, 1E11, that showed synergistic effects in combination with trastuzumab in gastric cancer cell lines (7). In another study, a mixture of three non-overlapping anti-HER2 antibodies showed a stronger anti-tumor activity than the combination of trastuzumab with pertuzumab *in vitro* and *in vivo* (8). It was also reported that a mAb targeting ErbB2 domain III provided synergy with either of trastuzumab or pertuzumab for inhibition of HER2 heterodimerization and cell signaling (9). GB235, another anti-HER2 mAb, in combination with trastuzumab inhibited tumor growth in the NCI-N87 gastric xenograft tumor model (10). There are other studies that reported similar results using different combinations of anti-HER2 mAbs (11).

Recently, we produced a humanized mAb, hersintuzumab, which targets domains I–II of human HER2, a distinct epitope from those recognized by trastuzumab and pertuzumab (12). Hersintuzumab induced growth inhibition, G1 cell cycle arrest, and cell signaling inhibition in HER2 expressing tumor cell lines (12). This mAb displayed significant synergistic inhibitory effects in combination with trastuzumab.

Bispecific antibodies (BsAbs) could be used to target two epitopes on the same or on distinct molecules instead of using two mAbs. So far, three BsAbs were approved in the USA and Europe (13), and many promising BsAbs are in the pipeline, targeting either two non-overlapping epitopes on HER2 (14–20) or HER2 together with other molecules such as HER3 (21, 22), CD3 (23–26), PD-L1 (27), 4-1BB (28), or CD63 (29).

Various molecular formats of BsAbs have been developed (30). Dual variable domain immunoglobulin (DVD-Ig) is composed of variable domains of two pre-existing mAbs *via* naturally occurring linkers, which yields a tetravalent IgG-like BsAb (31). In addition to a wide range of DVD-Igs directed against tumors (14, 16, 19, 32–34), several DVD-Igs targeting a variety of specificities such as TNF-α, IL-17A (35), IL-1α, and IL-1β (36) for treatment of autoimmune diseases, VEGF and PDGF for treatment of age-related macular degeneration (37), gp41 and gp120 for treatment of AIDS (38), TGF-β and fibronectin extra domain A (FN-EDA) for treatment of fibrotic kidneys (39), and amyloid-beta and transferrin receptor for treatment of Alzheimer’s disease (40) have been constructed and characterized recently. Some of these DVD-Igs are currently undergoing clinical trials (41–43).

In this study we generated and characterized a BsAb against HER2 in DVD-Ig platform, using variable domains of trastuzumab and hersintuzumab.

## Materials and Methods

### Cell Lines

Human breast cancer cell lines BT-474, MCF-7, JIMT-1, HCC-1954, human ovarian cancer cell line SKOV-3, human gastric cancer cell line NCI-N87 and CHO-K1 cells were obtained from the National Cell Bank of Iran (Pasture Institute of Iran, Tehran, Iran). All cell lines were cultured in RPMI 1640 medium (Gibco, Grand Island, NE, USA) supplemented with 100 U/mL penicillin, 100 μg/mL streptomycin (Gibco), and 10% fetal bovine serum (Gibco). BT-474 was cultured in 20% fetal bovine serum (Gibco) and also supplemented with 10 μg/mL human insulin (Exir Co., Boroojerd, Iran). 3T3-L1 was obtained from Iranian Biological Resource Center (Tehran, Iran) and was cultured in DMEM medium (Gibco) supplemented with 100 U/mL penicillin, 100 μg/mL streptomycin (Gibco), and 20% fetal bovine serum (Gibco).

### Design, Expression, Purification, and Structural Characterization of DVD-Igs

Two forms of anti-HER2 DVD-Igs, based on the proximity of variable domains of trastuzumab or hersintuzumab to N-terminal of the heavy and light chains of DVD-Ig, were designed as described previously (31). Briefly, the VL and VH sequences of hersintuzumab (12) and VL (GenBank: GM685466.1) and VH (GenBank: GM685464.1) sequences of trastuzumab were attached with two linkers ASTKGPSVFPLAP and TVAAPSVFIFPP, respectively, and then joined to human IgG1 heavy and Cκ light chain constant domains. These linkers are basically the same as the natural linkers present between the constant and variable domains of the heavy and light chains of an IgG molecule, providing a flexible conformation to both domains. These domains have been widely used in various DVD-Igs (33, 34, 44, 45). The genes of heavy and light chains of DVD-Igs were synthesized (Biomatik Co., Cambridge, ON, Canada) and cloned to a homemade dual-promoter expression plasmid that contains glutamine synthetase (GS) gene as a selection marker. The plasmid was then transfected to CHO-K1 cells by Lipofectamin 3000 reagent (Lifetechnology, USA) and stable clones were selected in the presence of methionine sulfoximine (MSX). The final selected high-producing clone was cultured in serum free medium and recombinant antibodies were purified with protein A affinity chromatography (GE Healthcare, USA). The purified DVD-Igs were run on 8% SDS-PAGE under reducing and non-reducing conditions. After electrophoresis, the gel was stained with Coomassie brilliant blue.

### Dual Specific Binding of DVD-Igs to Recombinant and Native Forms of HER2

For specific binding of each domain of DVD-Igs, recombinant full HER2-extracellular domain (ECD) and its subdomains, I+II and III+IV, which had been produced in our previous study (46), were coated in ELISA plate for 1.5 hours at 37°C. After 1 hour blocking with PBS containing 0.05% tween20 (PBS-T) and 3% skim milk (Merck, Darmstadt, Gemany) at 37°C, different concentrations of DVD-Igs or parental mAbs were added and incubated for 1 hour at 37°C and then HRP-conjugated rabbit anti human Ig (Sinabiotech Co., Tehran, Iran) was added and incubated for another 1 hour. After addition of TMB substrate (Pishtazteb Co., Tehran, Iran) at room temperature, the reaction was stopped with HCL 1N, and finally ODs were measured by an ELISA plate reader (Beckman Coulter, Brea, California, USA) at 450/630 nm.

Total IgG concentration was determined by an immunoglobulin-specific ELISA. Briefly, sheep anti-human Ig polyclonal antibody (SinaBiotech) was coated in 96 well-ELISA plate and blocked as previously described. Different concentrations of antibodies were then added and after incubation for 1 hour at 37°C and washing, HRP-sheep anti-human Ig polyclonal antibody (SinaBiotech) was added and the experiment proceeded as indicated above.

For competitive ELISA assay, recombinant full HER2-ECD or its subdomains, I+II and III+IV, were coated in ELISA plate for 1.5 hours at 37°C. After 1 hour blocking with PBS-T and 3% skim milk at 37°C, serial dilutions of DVD-Igs or parental mAbs were mixed with HRP-labeled trastuzumab or HRP-labeled hersintuzumab and incubated for an additional 1 hour at 37°C. After addition of TMB substrate at room temperature, the reaction was stopped with HCL 1N, and finally ODs were measured by an ELISA plate reader at 450/630 nm. The IC50 values of competitors were calculated using a four-variable algorithm.

For flow cytometry, different cell lines with various levels of HER2 expression were trypsinized and after twice washing with PBS, incubated with 100 µl of trastuzumab, hersintuzumab, or DVD-Igs at final concentration of 5 µg/ml on ice for 45 minutes. A chimeric anti-hepatitis B mAb harboring human IgG1/κ was used as isotype control (47). Cells were then washed and incubated with FITC-labeled sheep anti-human Ig antibody (Sinabiotech) on ice for additional 45 minutes, and after washing, cells were scanned by a flow cytometer (Partec, Nuremberg, Germany), and data were analyzed using the FlowJo v10 software.

For flow cytometry competitive binding assay, 1×10^6^ SKOV-3 cells were incubated with increasing concentrations of DVD-Igs, trastuzumab, or hersintuzumab and incubated for 45 minutes on ice. Then, FITC labeled trastuzumab or hersintuzumab (prepared in our laboratory) were added to the tubes and incubated on ice for another 45 minutes. After twice washing with PBS, cells were analyzed by flow cytometer. The IC50 values of competitors were calculated using a four-variable algorithm.

### Affinity Measurement

Binding affinity of DVD-Igs was measured with ELISA, as described previously (12). Briefly, serial concentrations (1200–75 ng/mL) of recombinant subdomains I+II, III+IV, or full HER2-ECD were coated in ELISA plate for 1.5 hours at 37°C. After 1 hour blocking with PBS-T containing 3% skim milk at 37°C, serial concentrations (1000–15.6 ng/mL) of DVD-Igs were added to the wells and incubated for 1 hour at 37°C. Then, HRP-conjugated rabbit anti-human Ig antibody was added and after 1 hour incubation at 37°C and washing twice with PBS-T, TMB substrate was added and after 5–10 min incubation at room temperature, the reaction was stopped with HCL 1N. Finally, the plate was read with plate reader at 450/630 nm. ODs were plotted against logarithmic values of antibody concentration and the antibody concentration giving 50% of the maximum absorbance value ([Ab]t) at a particular antigen coating concentration was chosen for the affinity measurement using the formula K_D_ = 1/2(2 [Ab’]t - [Ab]t). [Ab’]t and [Ab]t represents the antibody concentrations resulting in 50% of maximum absorbance value at two consecutive concentrations of coated antigen where [Ag] = 2[Ag’]. The mean of such calculations for three non-overlapping antigen concentrations was taken as the final K_D_ value.

### Proliferation Inhibition of Various Cancer Cell Lines by DVD-Igs

6×10^3^ BT-474, 2×10^3^ JIMT-1, 3.5×10^3^ HCC-1954, 1.5×10^3^ SKOV-3, and 11×10^3^ NCI-N87 cells were seeded in flat-bottomed 96-well plates and incubated overnight in a humidified atmosphere containing 5% CO2 at 37°C. DVD-Igs, hersintuzumab, trastuzumab (20, 1, 0.2, and 0.04 µg/ml) or combination of hersintuzumab and trastuzumab (10, 0.5, 0.1, and 0.02 µg/ml of each) were added to the wells, and after 3 days of incubation, 1 µCi3H–thymidine (PerkinElmer, MA, USA) was added to each well and incubated for an additional 20 hours. The cells were then harvested and transferred to the scintillation fluid and the amount of 3H-thymidine uptake was measured by a β-counter (Wallac 1410 Liquid Scintillation Counter, Pharmacia, Sweden). All experiments were performed at three independent times in triplicates. The proliferation inhibition rate was calculated with the use of following formula: [.

$$\begin{array}{cc} \text{Proliferation}\;\text{inhibition}(\%)=([\text{Counts}\;\text{per minute}(\text{CPM})\text{without}\;\text{antibody} & -\text{CPM}\;\text{with}\;\text{antibody}]/\text{CPM}\;\text{without}\;\text{anti body})\times 100 \end{array}$$

### Effect of DVD-Igs on Downstream Cell Signaling Pathways

Cell signaling studies were done as described previously (48). Briefly, BT-474, NCI-N87, SKOV-3, HCC-1954, JIMT-1, and MCF-7 cells were seeded in T-25 flasks and after an overnight incubation at 37°C in a humidified atmosphere containing 5% CO2, trastuzumab plus hersintuzumab or BiHT were added to the flask at a final concentration of 20 µg/ml and excipient was used as control. After 24 hours of incubation at 37°C and 5% CO2 atmosphere, cells were then trypsinized and lysed with M-PER reagent (Thermo Fisher Scientific, Waltham, Massachusetts, USA) containing Halt™ protease and phosphatase inhibitor (Thermo Fisher Scientific). Total protein content of cell lysates was normalized using BCA assay kit (Thermo Fisher Scientific) and were subjected to 10% SDS-PAGE, and after transferring to PVDF membrane, immunoblotting was done using antibodies against phosphorylated AKT, phosphorylated ERK, AKT, ERK, HER2, and β-actin as primary antibodies and HRP-conjugated anti-rabbit antibody (Cell signaling Technology, Danvers, Massachusetts, USA) as secondary antibody. These experiments were done once for all cell lines. Finally, protein bands were visualized with enhanced chemiluminescence prime kit (ECL, GE Healthcare, Uppsala, Sweden), scanned, and analyzed with ImageJ software. Band density was calculated using the following formula:

$$\text{Band density}=\left(\frac{\text{band density}\;\text{of}\;\text{mAb}}{\text{band density}\;\text{of}\;\text{its}\;\text{corresponding}\;\beta \text{Actin}}\right)/\left(\frac{\text{band density}\;\text{of}\;\mathrm{Control}\;(\text{excipient})}{\text{band density}\;\text{of}\;\text{its corresponding}\;\beta \text{Actin}}\right)$$

### Antibody-Dependent Cell-Mediated Cytotoxicity (ADCC)

A standard lactate dehydrogenase (LDH) release assay was performed to analyze ADCC. Normal human peripheral blood mononuclear cells (PBMCs) and SKOV-3 cells were used as effector and target cells respectively. Target cells were seeded in U-bottom 96-well plates at 10,000 cells/well and opsonized with serial dilutions of DVD-Igs and parental mAbs for 30 minutes. A chimeric anti-hepatitis B mAb harboring human IgG1/κ was used as isotype negative control (47). Then, PBMCs were added at 500,000/well and incubated in a humidified atmosphere containing 5% CO2 at 37°C for an additional 5 h. Then, 2% Triton-X 100 was used for maximum release of the LDH from target cells. The LDH released from cells in the culture supernatant was determined using a cytotoxicity assay kit (Promega, Madison, WI, USA). The percentage of cytotoxicity was calculated by the following formula:

Cytotoxicity % = (Experimental release − Effector cells spontaneous release − Target cells spontaneous release)/(Target cells maximum release − Target cells spontaneous release) × 100.

### Pharmacokinetics of BiHT

To determine pharmacokinetic properties, a single dose of 10 mg/kg of BiHT, hersintuzumab, or trastuzumab were injected intraperitoneally (IP) to 6-week-old female BALB/c mice. Trastuzumab was also injected intravenously (IV) as a control. Blood samples were taken from the lateral tail vein at 30 min, 2 h, and 1, 2, 7, 14, 21 days after administration and sera were collected and stored at -20°C. The antibody levels were measured by a sandwich ELISA. Briefly, mouse mAb specific to human IgG1 (Sinabiotech) were coated overnight in an ELISA plate at 4°C and after 1 hour blocking with PBS-T containing 3% skim milk at 37°C, serial dilutions of mouse sera were added into the wells. Then, mouse-adsorbed HRP-conjugated rabbit anti-human Ig (Sinabiotech) were added and after 1 hour of incubation at 37°C and three times washing with PBS-T, TMB substrate was added and the reaction was then stopped with 1N HCL. Finally, the ODs were measured with a plate reader at 450/630 nm. Pharmacokinetic parameters were calculated by a noncompartmental analysis using Phoenix Winnonlin (31).

### Tumor Growth Inhibition *In Vivo*

Two HER2 over-expressing cell lines were employed for xenograft study. NCI-N87 cells (5×10^6^ in Matrigel; Corning, Life Sciences, Corning, NJ) and SKOV-3 cells (5×10^6^ in PBS) were implanted subcutaneously into the right flank of female BALB/c nude mice. When the tumor volume reached about 100–150 mm^3^, the mice were randomly divided in three groups of 6 mice. DVD-Ig (10mg/kg), Trastuzumab plus Hersintuzumab (5 mg/kg of each), or PBS were intraperitoneally injected once a week for 6 consecutive weeks. Tumor sizes were measured three times a week and the tumor volumes were calculated by the formula: volume = length × (width)^2^/2. All animal experiments were performed according to the permission granted by the Ethics Committee of Tehran University of Medical Sciences (IR NIMAD REC 1396 060).

### Statistics

The results were analyzed with One-Way ANOVA test using SPSS software (version 20, IBM SPSS statistics data editor). Differences between groups were significant at p values less than 0.05 (p < 0.05). The results are presented as mean ± standard error of the mean (SEM).

## Results

### Design, Construction, Expression, and Characterization of Anti-HER2 DVD-Igs

**Figure 1:**
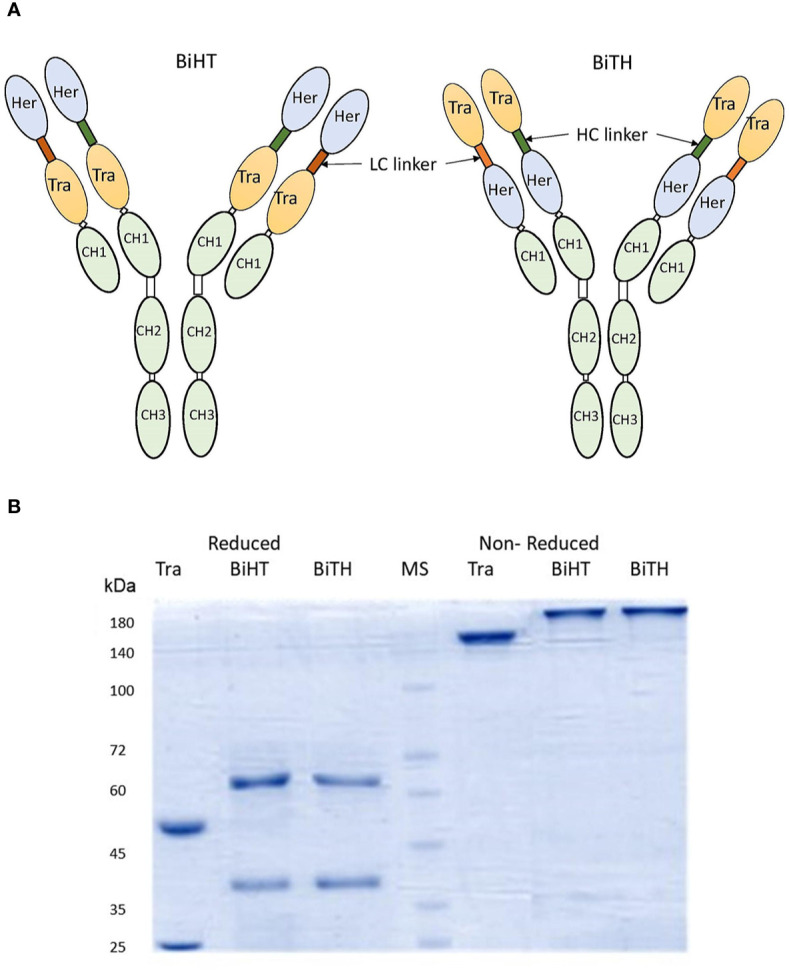
Structural characterization of anti-HER2 DVD-Igs. **(A)** Schematic presentation of BiHT and BiTH design. **(B)** SDS-PAGE analysis of trastuzumab, BiHT and BiTH under reducing (left) and non-reducing (right) conditions. Tra, trastuzumab; Her, hersintuzumab; HC, heavy chain; LC, light chain; MS, molecular size.

Two DVD-Igs (BiHT and BiTH) were engineered using variable domains of trastuzumab and hersintuzumab, which are joined together in tandem with different orientations (**Figure 1A**). Both DVD-Igs were expressed in conjunction with human IgG1 and Cκ constant domains. The structure of DVD-Igs were analyzed by SDS-PAGE, and the results showed similar patterns for both DVD-Igs with a monomeric form (200 kDa) under nonreducing and two monomeric heavy (~65 kDa) and light (~40 kDa) chains under reducing conditions (**Figure 1B**).

**Figure 2:**
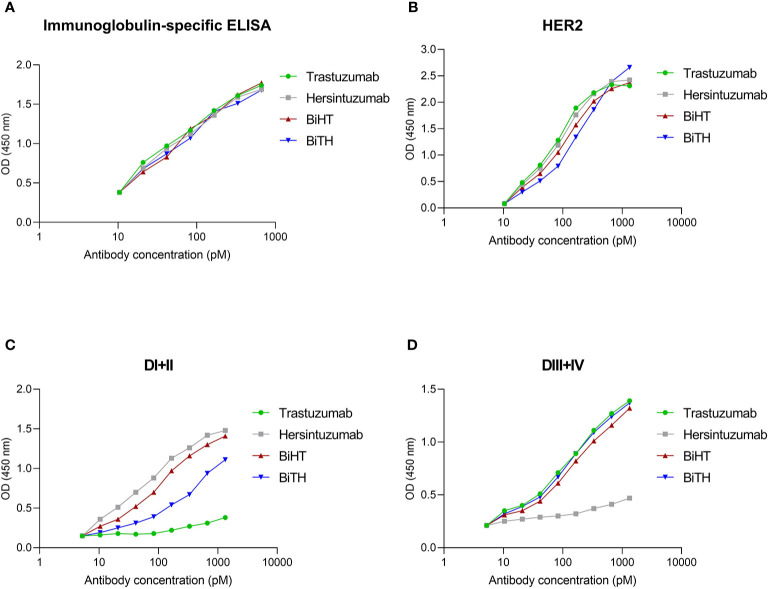
The profile of binding of DVD-Igs and their parental mAbs to HER2 and its subdomains. Different concentrations of antibodies were comparatively determined by an immunoglobulin-specific ELISA **(A)**. Binding patterns of trastuzumab, hersintuzumab, BiHT, and BiTH with full HER2-ECD and its subdomains DI+II and DIII+IV were analyzed by a specific ELISA **(B–D)**.

The constructs were transfected in CHO cells, and the BsAbs were screened in culture supernatant by ELISA. Immunoglobulin-specific ELISA was used to determine the concentration of each antibody (**Figure 2A**), and the same molar concentration of DVD-Igs and mAbs were used in all experiments. Dual specific binding and affinity of DVD-Igs to subdomain I+II (hersintuzumab epitope), subdomain III+IV (trastuzumab epitope), and full ECD of human HER2 were assessed by a HER2-specific ELISA. The results demonstrated that both DVD-Igs were able to bind specifically to their epitopes. The binding profiles of both BiHT and BiTH to full ECD and subdomain III+IV at different concentrations were quite similar to those of the parental mAbs (**Figure 2B**). However, while reactivity of BiHT to subdomain I+II was similar to hersintuzumab, reactivity of BiTH to subdomain I+II was notably weaker than that of the parental hersintuzumab (**Figures 2C, D**).

The affinity constant of each BsAb to HER2-ECD was similar to the mean of affinity constants of the parental mAbs (**Table 1**). The affinity constant of BiHT to DI+II and DIII+IV (i.e., the target of its inner and outer variable domains, respectively) have decreased 2–3 times, as compared to the corresponding parental mAbs (**Table 1**). However, while the affinity constant of BiTH to DIII+IV (i.e., the target of its outer variable domain) was similar to that of trastuzumab, its affinity to DI+II (i.e., the target of its inner variable domain) has decreased 11 times as compared to the corresponding parental mAb, hersintuzumab (**Table 1**). In other words, the variable domain of trastuzumab is more compatible with the inner orientation of DVD-Ig, as compared to hersintuzumab.

**Table 1:** Affinity constant of bispecific mAbs and the parental antibodies to full HER2-ECD and its subdomains.

Antibodies	K_D_ (DI+II) nM	K_D_ (DIII+IV) nM	K_D_ (ECD) nM
Trastuzumab	–	0.59	0.84
Hersintuzumab	0.13	–	0.22
BiHT	0.43	1.37	0.61
BiTH	1.41	0.38	0.50

**Figure 3:**
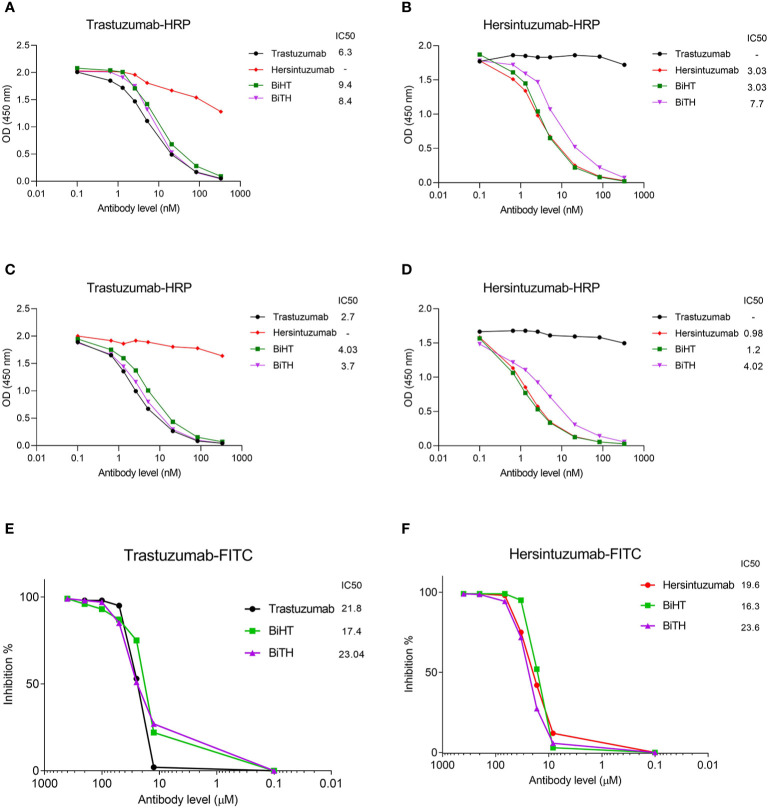
Binding competition and related IC50 of BsAbs. Trastuzumab, hersintuzumab, BiHT, and BiTH compete with trastuzumab-HRP **(A, C)** or hersintuzumab-HRP **(B, D)** for binding to recombinant HER2-ECD **(A, B)**, and subdomains III+IV **(C)** and I+II **(D)** by ELISA. Binding competition of trastuzumab, hersintuzumab, BiHT, and BiTH with FITC-labeled trastuzumab **(E)** and FITC-labeled hersintuzumab **(F)** to native HER2 on SKOV-3 cell line was also assessed by flow cytometry. IC50: The half maximal inhibitory concentration.

Competitive binding assays were used to assess the relative binding affinity of BiHT and BiTH for their epitopes expressed on recombinant HER2-ECD, subdomain I+II, or subdomain III+IV, using ELISA (**Figures 3A–D**) and flow cytometry (**Figures 3E, F**). The results showed that IC50 values of BiHT for recombinant HER2-ECD (**Figure 3A**), subdomain III+IV (**Figure 3C**) and native HER2 (**Figures 3E, F**) were similar to the parental mAbs. Despite the slight decrease in the IC50 values in competitive ELISA (**Figures 3B, D**), BiTH retained the full binding activity for native HER2 in competitive flow cytometry (**Figures 3E, F**).

**Figure 4:**
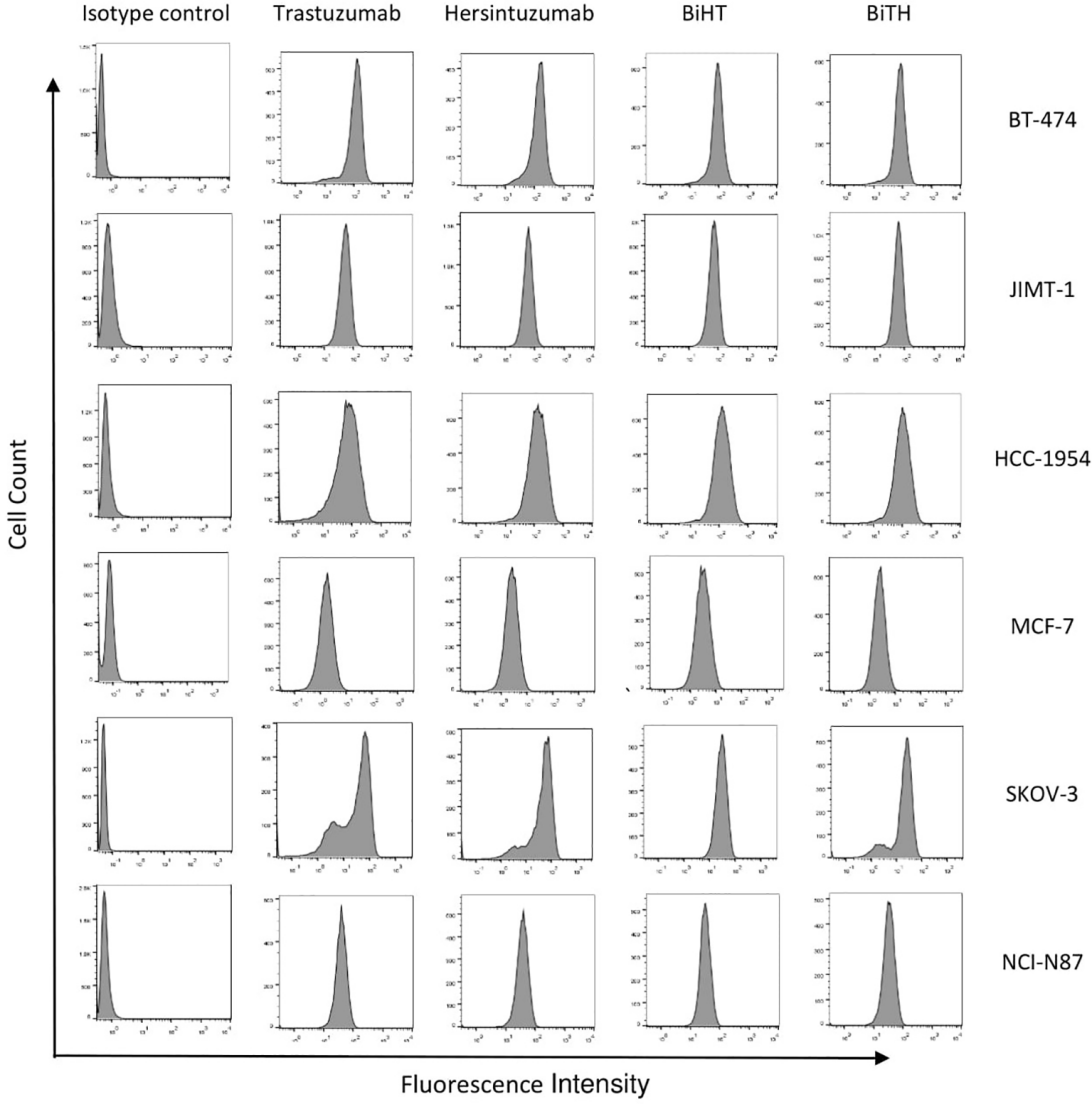
Binding profile of DVD-Igs and their parental antibodies to HER2-expressing tumor cell lines. BT-474, JIMT-1, HCC-1954, MCF-7, SKOV-3, and NCI-N87 cell lines were treated with trastuzumab, hersintuzumab, BiHT, BiTH as primary antibodies and then FITC labeled anti-human Ig polyclonal antibody was used as the secondary antibody. The final results were analyzed by flow cytometry. A chimeric anti-hepatitis B mAb harboring human IgG1/κ was used as isotype control.

In order to determine the binding profile of DVD-Igs to the HER2-overexpressing tumor cells, flow cytometry analysis was conducted. Both DVD-Igs could detect HER2 on the surface of different cancer cell lines similar to trastuzumab and hersintuzumab with regards to percent of positive cells and mean fluorescence intensity (**Figure 4**). Taking together, results of binding assays demonstrate that BiHT retains full binding activity to both of the corresponding epitopes in comparison to the parental mAbs. However, the binding activity of BiTH is decreased as indicated by the competitive ELISA results (**Figures 3B, D**).

### Effects of Bispecific Antibodies on Tumor Cells Proliferation

**Figure 5:**
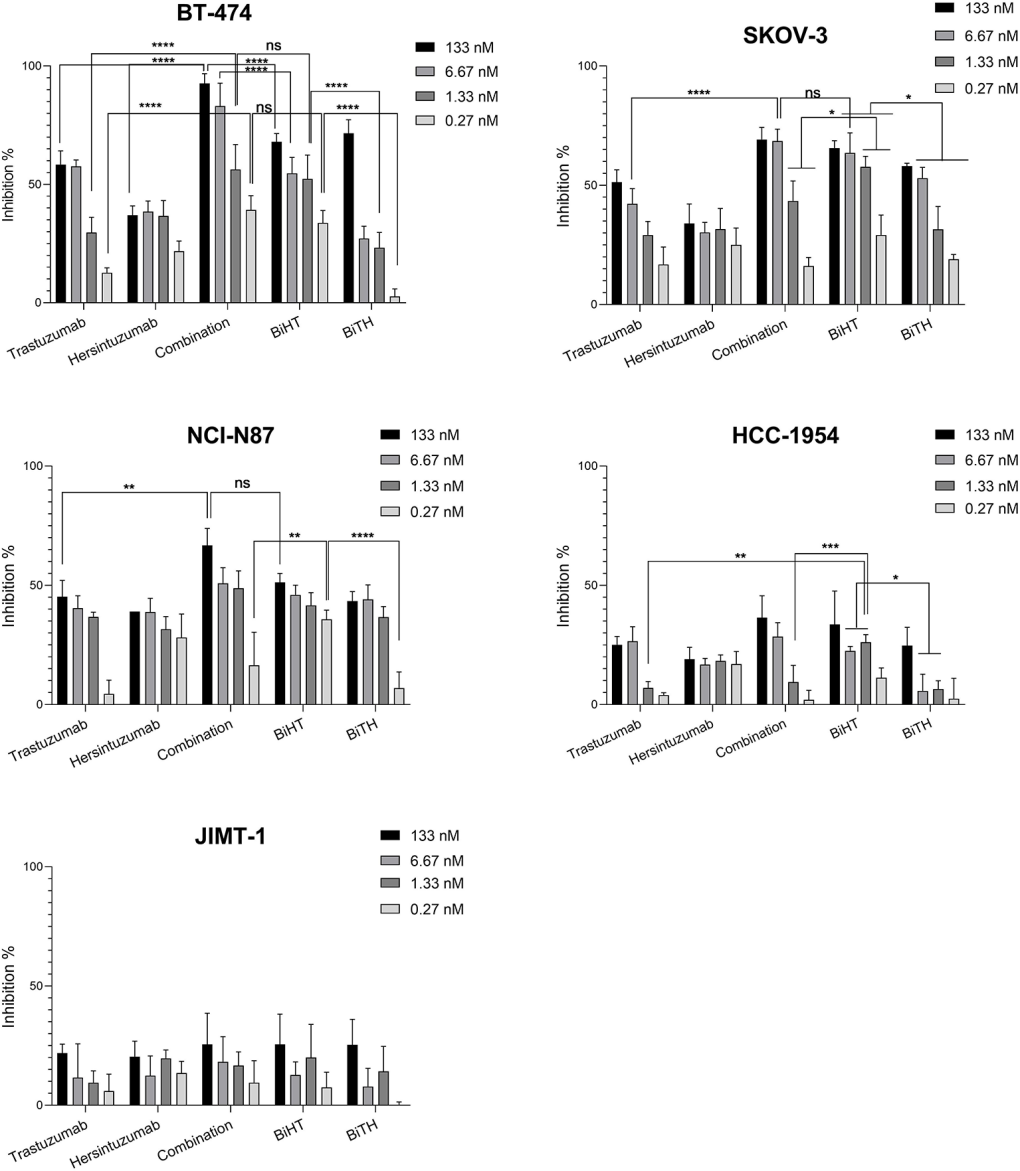
Anti-proliferative effects of BsAbs and their parental mAbs on the proliferation of BT-474, HCC-1954, JIMT-1, SKOV-3, and NCI-N87 cell lines. Data represent percentage of cell growth inhibition of triplicate cultures conducted three times. Combination denotes treatment with trastuzumab and hersintuzumab at a half dose of the indicated concentration for each mAbs. ****P < 0.0001, ***P < 0.001, **P < 0.01, *P < 0.05, ns: non-significant.

^3^H-thymidine incorporation assay was used to assess the antiproliferative effect of DVD-Igs on BT-474, SKOV-3, NCI-N87, and the trastuzumab resistant cell lines, HCC-1954 and JIMT-1. Trastuzumab, hersintuzumab, and their combinations were used as controls. The results indicated that both anti-HER2 DVD-Igs and the combination of trastuzumab and hersintuzumab inhibited the proliferation of BT-474, SKOV-3, and NCI-N87 more significantly than each parental mAb alone, at all treatment doses (**Figure 5** and **Supplementary Table 1**). Interestingly, at lower doses, the antiproliferative effect of BiHT was more potent than BiTH as well as the combination of trastuzumab and hersintuzumab in SKOV-3, NCI-N87, and BT-474 cell lines. Similar results were obtained with the trastuzumab resistant cell line HCC-1954. At higher doses, the antiproliferative effect of BiHT was as potent as the combination of trastuzumab and hersintuzumab in all cell lines with the exception of BT-474, in which the combination was more potent than BiHT and BiTH (**Figure 5** and **Supplementary Table 1**). However, JIMT-1, the other trastuzumab resistant cell line, displayed a different profile, and the antibodies showed poor antiproliferative effect on this cell line.

### Antibody-Dependent Cell-Mediated Cytotoxicity (ADCC)

**Figure 6:**
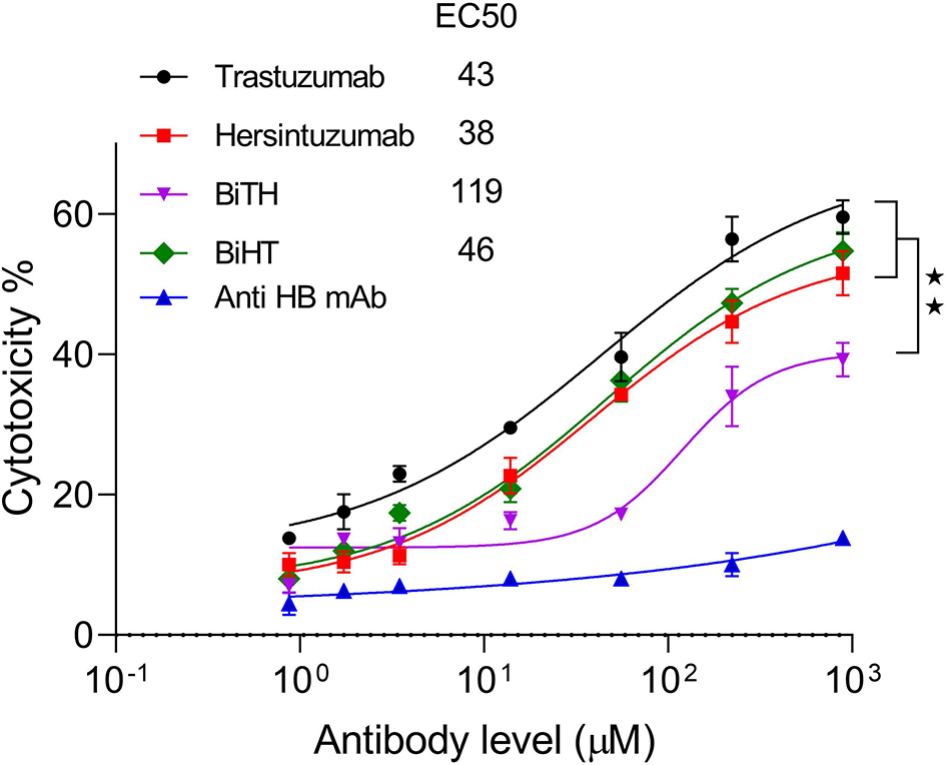
Antibody dependent cell cytotoxicity (ADCC) of the BsAbs. SKOV-3 cells were cocultured with human PBMCs in the prescence of serial concentrations of mAbs and LDH release was measured in the supernatants. A chimeric anti-hepatitis B mAb harboring human IgG1/κ was used as isotype negative control. The ADCC experiments were performed in triplicate, and the data are representative of mean ± SEM of two independent experiments. EC50: The half maximal effective concentration, HB: hepatitis B, **P < 0.01.

LDH release assay was performed using normal human PBMCs as effector cells and SKOV-3 tumor cells as target cells. The results showed that ADCC activity of BiHT was comparable to those of trastuzumab and hersintuzumab. However, ADCC activity of BiTH was decreased to a quarter of the parental mAbs (**Figure 6**). According to the results of the proliferation and ADCC assays, we decided to select BiHT for the rest of the experiments.

### Pharmacokinetics Analysis

**Figure 7:**
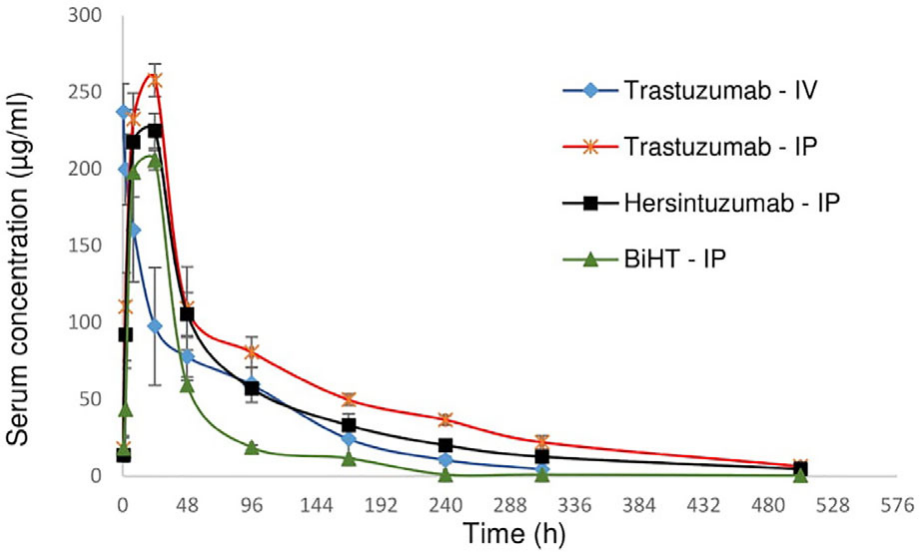
Pharmacokinetics profile of BiHT, trastuzumab, and hersintuzumab. Serum concentrations (± SEM) were measured at different time intervals after administration of a single dose of 10 mg/kg (intraperitoneally/IP or intravenously/IV) in BALB/c mice (3 mice/group). IP: intraperitoneal, IV: intravenous.

In order to study the pharmacokinetics of BiHT, trastuzumab, and hersintuzumab, 12 female mice (three in each group) were administered intraperitoneally (IP) with BiHT, trastuzumab, and hersintuzumab and intravenously with trastuzumab alone. The concentration of each antibody was measured in serum at different time intervals by a sandwich ELISA. As demonstrated in **Figure 7** and **Table 2**, the pharmacokinetic parameters of BiHT were similar to those of the parental antibodies, indicating that this BsAb is probably stable *in vivo*, although stability needs to be confirmed with other experiments such as Western blotting. In order to substantiate our pharmacokinetic results and compare them with the results reported in the literature, since most papers have employed a single IV administration of trastuzumab, we decided to include a single IV administration of trastuzumab in our study. Our data are compatible with those reported by other investigators (14). Since cross reactivity of the anti-human HER2 mAbs with murine HER2 affects the pharmacokinetics of these mAbs, we checked cross reactivity of hersintuzumab and transtuzumab with mouse HER2 by flow cytometry, using a HER2 expressing mouse cell line (3T3-L1). No cross reactivity was observed (**Supplementary Figure 1**).

**Table 2:** Pharmacokinetic parameters of BiHT, trastuzumab, and hersintuzumab in BALB/c mice (3 mice/group).

		Trastuzumab	Hersintuzumab	BiHT
IP	Tmax (h)	24	24	24
	Cmax (µg/ml)	258 ± 18	225 ± 24	206 ± 2
	T1/2 (h)	124.8 ± 7	105 ± 6	82 ± 4
	Clearance (L/h)	0.004	0.005	0.005
IV		Trastuzumab		
	Clearance (L/h)	0.006		
	T1/2 (h)	119.04 ± 5		

Tmax, time point of maximum concentration of the antibodies; Cmax, maximum concentration of the antibodies; T1/2, half-life; IP, intraperitoneal; IV, intravenous.

### Inhibition of Cell Signaling and HER2 Downregulation

**Figure 8:**
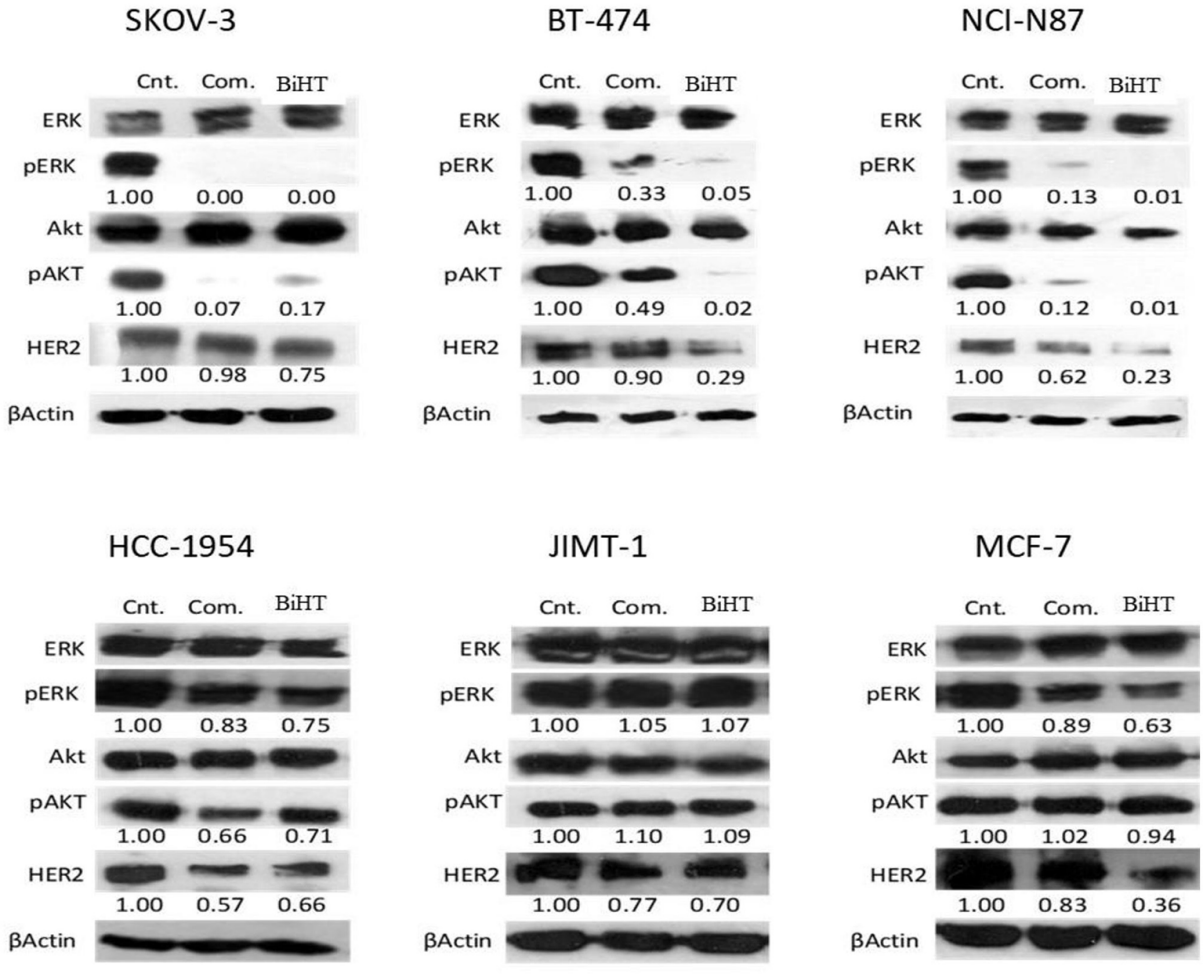
Inhibitory effects of BiHT on phosphorylation of the AKT and ERK1/2 signaling pathways and HER2 expression. BT-474, SKOV-3, NCI-N87, HCC-1954, JIMT-1, and MCF-7 cell lines were treated with BiHT (20 µg/ml) or combination of trastuzumab and hersintuzumab (10 µg/ml, each) for 24 h. Cell lysates were separated on SDS-PAGE and immunoblotted to detect AKT, pAKT, ERK1/2, pERK1/2, HER2, and beta actin as a house keeping protein. Cnt: Control, Com: Combination. The figures presented underneath of each mAb treatment represent the band densities calculated as described in Materials and Methods.

MAPK and AKT/PI3K are the two major signaling pathways affected by the HER family. We evaluated the effects of BiHT and trastuzumab plus hersintuzumab on the phosphorylation of these signaling pathways in BT-474, SKOV-3, NCI-N87, HCC-1954, JIMT-1, and MCF-7 cell lines. The results showed that BiHT induced almost complete inhibition of phosphorylation of AKT and ERK1/2 in BT-474, SKOV-3, and NCI-N87, while it slightly suppressed phosphorylation of AKT and ERK in HCC-1954 and ERK in MCF-7, but failed to inhibit AKT/ERK signaling pathways in JIMT-1 cell line. All these inhibitory effects were similar to those obtained by the combination of parental antibodies, with the exception of BT-474 and NCI-N87, which displayed more potent phosphorylation inhibition of AKT/ERK by BiHT than the combination of trastuzumab and hersintuzumab. The combination therapy was slightly more potent than BiHT in the phosphorylation inhibition of AKT in SKOV-3 (**Figure 8**). Interestingly, BiHT downregulated HER2 expression more efficiently than the combination of the two parental mAbs in almost all cell lines, except for JIMT-1 and HCC-1954.

### Growth Suppression of Ovarian and Gastric Cancer Xenografts in Nude Mice

**Figure 9:**
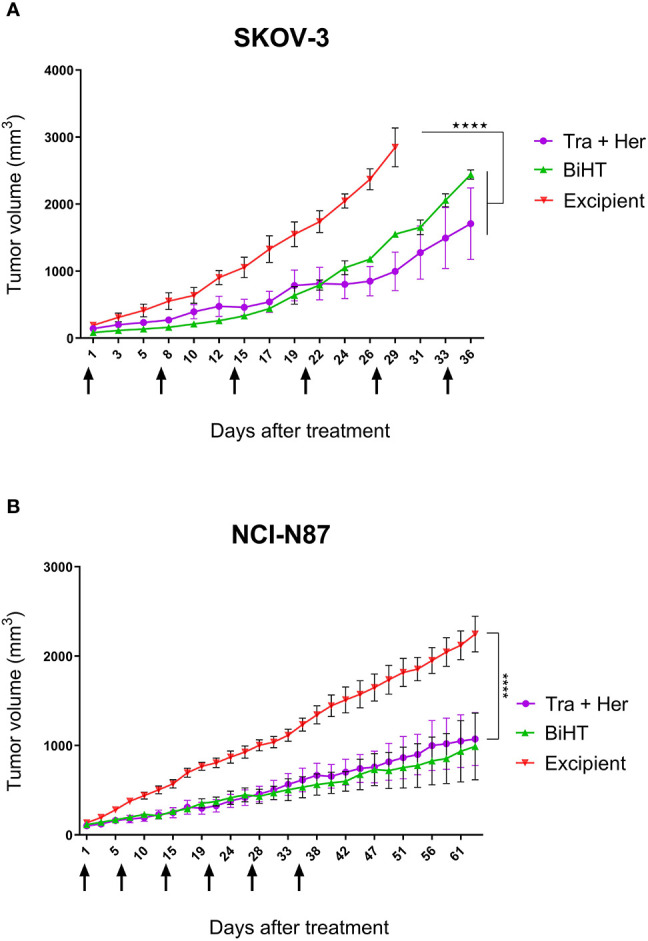
*In vivo* therapeutic efficacy of BiHT mAb in nude mice. SKOV-3 **(A)** and NCI-N87 **(B)** xenograft tumor bearing nude mice were treated once a week with a combination of trastuzumab plus hersintuzumab (5 mg/kg of each), or BiHT (10 mg/kg), for 6 consecutive weeks and the control group received excipient alone. Tumor sizes were measured three times a week. Data is shown as mean ± SEM. ****P < 0.0001. Tra: trastuzumab. Her: hersintuzumab. Arrows show timing of mAb administration.

The potential therapeutic efficacy of BiHT and the combination of trastuzumab and hersintuzumab was determined in nude BALB/c mice bearing established SKOV-3 and NCI-N87 xenografts. The results in SKOV-3 xenograft indicate that mice treated with BiHT and the combination of the two parental mAbs had significantly lower tumor size than the control group (excipient). Mice treated with the combination of mAbs displayed relatively smaller tumor sizes than the BiHT group, but the differences were statistically insignificant (**Figure 9**). Similar results were obtained in NCI-N87 xenograft treated mice. Tumor sizes of mice treated with BiHT and the combination of mAbs were highly similar to each other and significantly lower than those of the control group (**Figure 9**). Moreover, we have previously shown in a pilot study that the combination of trastuzumab and hersituzumab was more potent than each antibody alone for regression of both NCI-N87 and SKOV-3 xenograft tumors (**Supplementary Figure 2**).

## Discussion

Despite the great success of mAbs in cancer therapy, many efforts are being made to improve their therapeutic efficacy. Combination therapy of two mAbs is a promising strategy to achieve synergistic effects of anti-cancer therapy. Combination of trastuzumab and pertuzumab disrupted the heterodimerization of HER2 with HER3, inhibited AKT signaling in BT-474, and suppressed HER2-positive breast and non-small cell lung cancer xenografts more potently than either monotherapy (49, 50). Many research groups try to develop novel anti-HER2 mAbs hoping to use them in combination with another anti-HER2 mAb to obtain better inhibitory synergistic effects (7–9).

In our previous work, we developed a new humanized mAb designated hersintuzumab, which recognizes different epitope than trastuzumab and pertuzumab without any cross-reactivity with other members of the HER family (US patent: US20170066829A1). Preliminary functional characterization of this mAb indicated that it induces cell cycle arrest at G1 phase and in combination with trastuzumab had more potent antiproliferative effect on tumor cell lines than the combination of pertuzumab and trastuzumab (12). Moreover, its combination with trastuzumab significantly inhibited both AKT and ERK phosphorylation more potently than the combination of trastuzumab and pertuzumab (48). In accordance with *in vitro* studies, hersintuzumab in combination with trastuzumab showed superior anti-tumor activity than each mAb alone in ovarian and gastric xenograft bearing mice (**Supplementary Figure 2**), which makes it a perfect partner for generation of a new BsAb antibody.

Converting non-overlapping and non-competitive mAbs to a BsAb antibody enables us to have synergistic effects of two mAbs in one molecule (30). BsAb antibodies that bind two different epitopes may have synergistic effects on different aspects of HER2 functions, such as heterodimerization and cell signaling. At least three research groups developed anti-HER2 BsAbs from trastuzumab and pertuzumab in different formats (14, 16, 17). In this study, we used DVD-Ig technology (31) to develop two BsAbs, designated BiHT and BiTH, using variable domains of trastuzumab and our new anti-HER2 mAb, hersintuzumab, which bind to the domain IV and I+II of human HER2-ECD, respectively. We used a long linker between variable domains of trastuzumab and hersintuzumab in the light and heavy chains of BiHT and BiTH. The length of the linkers between two tandem variable domains of a DVD-Ig has been shown to affect the affinity of the inner domain, long linkers display better flexibility and binding affinity than short linkers for some variables domain of mAbs (44, 45, 51, 52). Another consideration in the design of DVD-Ig is the orientation of the two variable domains (53). Accordingly, we generated BiHT and BiTH, in which hersintuzumab is in the outer and trastuzumab is in the inner variable domain and vice versa, respectively.

Both DVD-Igs were transfected and expressed in CHO-K1 cells without any intermediate light or heavy chain surplus byproduct after purification and they retained their IgG like structure (**Figure 1B**). We found that the binding affinity of trastuzumab variable domain in BiHT and BiTH is similar to the corresponding parental mAb, trastuzumab. Moreover, binding affinity of hersintuzumab variable domain in BiTH is far less than that of BiHT (**Table 1**). In fact, when trastuzumab variable domain is in the outer or inner domain of DVD-Ig, its reactivity doesn’t change profoundly. In contrast, the reactivity of hersintuzumab variable domain decreases in the inner, but not in the outer position. In accordance with our results, Wu and colleague showed that IL-1a variable domain retains potency of parental mAb, even with short linker, in the inner and outer domains of DVD-Ig. In contrast, potency of IL-1b variable domain reduces in the inner domain of DVD-Ig and it seems that IL-1a variable domain is more compatible with the inner domain position than IL-1b variable domain (44). Bohua et al. showed that with the use of a long linker, trastuzumab keeps its potency either in the inner or outer layer of DVD-Igs, but not with a short linker (14). In another study it was demonstrated that trastuzumab retains its activity in both orientations, even with a short linker (16).

The results obtained from ELISA and flow cytometry (**Figures 3C, D**) indicated that despite the decrease in the binding activity of BiTH to subdomain I+II (**Figure 2C**), there is no significant difference between BiHT and BiTH for binding to recombinant full HER2-ECD and native HER2 on the surface of different cell lines (**Table 1** and **Figure 4**), which might be due to the compensatory binding activity of the variable domain of trastuzumab. None of the previous works used subdomains of HER2 to determine the reactivity and affinity of their BsAbs (14, 16).

Antiproliferative effect of BiHT on different HER2-overxpressing cell lines showed that with the exception of the JIMT-1 cell line, it inhibits the growth of all cell lines doses dependently, similar to the combination of the parental mAbs. At subsaturated doses, BiHT showed more potent growth inhibitory effects than the combination of the two parental mAbs or BiTH. In contrast to BiTH, the ADCC activity of BiHT was similar to the parental mAbs. Significantly lower ADCC activity of BiTH compared to BiHT could be associated with its lower HER2 binding affinity. The effect of antigen binding affinity on ADCC activity has already been reported for other antibody specificities (17, 54, 55). As a result, we omitted BiTH from the rest of the experiments and focused on BiHT. The growth inhibitory results were in accordance with the results of downstream signaling pathways, showing significant inhibitory of phosphorylation of AKT and ERK by BiHT treatment (**Figure 8**). Jinming Gu et al. developed eight anti-HER2 DVD-Igs from trastuzumab and pertuzumab with different orientations of variable domains and different linker sizes. Four of these DVD-Igs inhibited the cell proliferation of NCI-N87 cells while the other four DVD-Igs enhanced the proliferation of this cell line (16). Enhancement or inhibition of cell proliferation by these two groups of BsAb was attributed to the avidity, together with domain orientation of DVD-Igs, which may induce different HER2 conformational changes. Both of our DVD-Igs displayed anti-proliferative effects in four cell lines from three different tissue origins.

In ovarian and gastric cancer cell lines, SKOV-3 and NCI-N87, BiHT suppressed xenograft tumor growth as efficiently as the combination of trastuzumab and hersintuzumab (**Figure 9**). In our pilot study we observed that the combination of trastuzumab and hersintuzumab was significantly more effective than each mAb alone for the inhibition of SKOV-3 and NCI-N87 xenograft models (**Supplementary Figure 2**).

As we can see from the proliferation experiments of SKOV-3 and NCI-N87 cell lines (**Figure 5**), there is little difference between 1 and 20 µg/ml of mAb concentration for proliferation inhibition, which indicates that 1 µg/ml is a saturated concentration. Moreover, in the pharmacokinetics experiments, serum concentration of all administrated mAbs is more than 1 µg/ml one week after 10 mg/kg IP or IV administration (**Figure 7**). Based on these considerations we decided to use 10 mg/kg of the combination of trastuzumab and hersintuzumab or BiHT once a week in our xenograft model. Harumi Sakahara et al. demonstrated that the concentration of the antibodies in the tissues expressing the specific ligand is 1.2–17 times more than their serum concentration after 96 hours of injection (56). As a consequence, it could be deduced that the dose of the antibodies in the tumor niche of our xenograft model is much more than the saturation dose and similar to the proliferation experiments, there is no difference between BiHT and the combination of trastuzumab and hersintuzumab at this dose.

Altogether, our results demonstrated that BiHT was as efficient as the combination of the parental mAbs *in vitro* and *in vivo*. As a result, having the additive or synergistic effects of the two mAbs in one BsAb reduces drug cost due to manufacturing of a single mAb instead of two mAbs, improves safety of patients due to less manipulation, and increases patients’ satisfaction.

## Conclusion

In this study we demonstrated that our novel BsAb (BiHT), designated trasintuzumab, is a dual functional and *in vivo* stable IgG1 antibody, which inhibits cell proliferation, downregulates HER2 expression, and blocks AKT and ERK downstream signaling pathways. It displayed anti-tumor activity *in vitro* and *in vivo* as efficiently as the combination of the parental mAbs trastuzumab and hersintuzumab. Trasintuzumab is expected to exhibit more potent therapeutic effects than the combination of trastuzumab and pertuzumab and is a potential therapeutic candidate for treatment of HER2 overexpressing cancer types.

## Data Availability Statement

The raw data supporting the conclusions of this article will be made available by the authors, without undue reservation.

## Ethics Statement

The animal study was reviewed and approved by ethics committee of National Institute for Medical Research Development of Iran - NIMAD (IR NIMAD REC 1396 060).

## Author Contributions

FS and MMA made substantial contributions to the study conception, design, analysis and interpretation of the data. MM made substantial contributions to the design, acquisition, analysis and interpretation of the data. MA, TB, HZ, FH, FS, MAJ and MM contributed to the acquisition of the data. The first draft of the manuscript was written by MM and MMA. FS, FG-S and MJ-T commented on subsequent versions of the manuscript. All authors contributed to the article and approved the submitted version. FS is the guarantor for the overall content of the article.

## Funding

This study was partially supported by grants from National Institute for Medical Research Development of Iran (Grant No. 957625), Tehran University of Medical Sciences (Grant No. 97-02-27-38122), Avicenna Research Institute (Grant No. 97-004), and Academic Center for Education, Culture and Research/ACECR (Grant No. 3075-33).

## Conflict of Interest

The authors declare that the research was conducted in the absence of any commercial or financial relationships that could be construed as a potential conflict of interest.

## References

[1] Graus-Porta D Beerli RR Daly JM Hynes NE. ErbB-2, the preferred heterodimerization partner of all ErbB receptors, is a mediator of lateral signaling. EMBO J (1997) 16(7):1647–55. 10.1093/emboj/16.7.1647 PMC1169769 9130710

[2] Citri A Yarden Y. EGF–ERBB signalling: towards the systems level. Nat Rev Mol Cell Biol (2006) 7(7):505–16. 10.1038/nrm1962 16829981

[3] Tai W Mahato R Cheng K. The role of HER2 in cancer therapy and targeted drug delivery. J Controlled Release (2010) 146(3):264–75. 10.1016/j.jconrel.2010.04.009 PMC2918695 20385184

[4] Hudis CA. Trastuzumab–mechanism of action and use in clinical practice. N Engl J Med (2007) 357(1):39–51. 10.1056/NEJMra043186 17611206

[5] Adams CW Allison DE Flagella K Presta L Clarke J Dybdal N. Humanization of a recombinant monoclonal antibody to produce a therapeutic HER dimerization inhibitor, pertuzumab. Cancer Immunology Immunotherapy (2005) 55(6):717. 10.1007/s00262-005-0058-x 16151804 PMC11030689

[6] Baselga J Cortes J Kim SB Im SA Hegg R Im YH. Pertuzumab plus trastuzumab plus docetaxel for metastatic breast cancer. N Engl J Med (2012) 366(2):109–19. 10.1056/NEJMoa1113216 PMC5705202 22149875

[7] Ko B-K Lee S-Y Lee Y-H Hwang I-S Persson H Rockberg J. Combination of novel HER2-targeting antibody 1E11 with trastuzumab shows synergistic antitumor activity in HER2-positive gastric cancer. Mol Oncol (2015) 9(2):398–408. 10.1016/j.molonc.2014.09.007 25306393 PMC5528653

[8] Pedersen MW Jacobsen HJ Koefoed K Dahlman A Kjær I Poulsen TT. Targeting three distinct HER2 domains with a recombinant antibody mixture overcomes trastuzumab resistance. Mol Cancer Ther (2015) 14(3):669–80. 10.1158/1535-7163.MCT-14-0697 25612619

[9] Meng Y Zheng L Yang Y Wang H Dong J Wang C. A monoclonal antibody targeting ErbB2 domain III inhibits ErbB2 signaling and suppresses the growth of ErbB2-overexpressing breast tumors. Oncogenesis (2016) 5:e211. 10.1038/oncsis.2016.25 26999718 PMC4815051

[10] Shu M Yan H Xu C Wu Y Chi Z Nian W. A novel anti-HER2 antibody GB235 reverses Trastuzumab resistance in HER2-expressing tumor cells in vitro and in vivo. Sci Rep (2020) 10(1):e2986. 10.1038/s41598-020-59818-2 PMC7031383 32076029

[11] Tahmasebi F Kazemi T Amiri MM Khoshnoodi J Mahmoudian J Bayat AA. In vitro assessment of the effects of anti-HER2 monoclonal antibodies on proliferation of HER2-overexpressing breast cancer cells. Immunotherapy (2013) 6(1):43–9. 10.2217/imt.13.156 24341883

[12] Amiri MM Golsaz-Shirazi F Soltantoyeh T Hosseini-Ghatar R Bahadori T Khoshnoodi J. Hersintuzumab: A novel humanized anti-HER2 monoclonal antibody induces potent tumor growth inhibition. Invest New Drugs (2018) 36(2):171–86. 10.1007/s10637-017-0518-0 28983766

[13] Labrijn AF Janmaat ML Reichert JM Parren PWHI. Bispecific antibodies: a mechanistic review of the pipeline. Nat Rev Drug Discov (2019) 18(8):585–608. 10.1038/s41573-019-0028-1 31175342

[14] Li B Meng Y Zheng L Zhang X Tong Q Tan W. Bispecific antibody to ErbB2 overcomes trastuzumab resistance through comprehensive blockade of ErbB2 heterodimerization. Cancer Res (2013) 73(21):6471–83. 10.1158/0008-5472.can-13-0657 24046294

[15] Brack S Attinger-Toller I Schade B Mourlane F Klupsch K Woods R. A bispecific HER2-targeting FynomAb with superior antitumor activity and novel mode of action. Mol Cancer Ther (2014) 13(8):2030–9. 10.1158/1535-7163.mct-14-0046-t 24994770

[16] Gu J Yang J Chang Q Lu X Wang J Chen M. Identification of Anti-ErbB2 Dual Variable Domain Immunoglobulin (DVD-Ig™) Proteins with Unique Activities. PLoS One (2014) 9(5):e97292. 10.1371/journal.pone.0097292 24824849 PMC4019538

[17] Zhang F Zhang J Liu M Zhao L LingHu R Feng F. Combating HER2-overexpressing breast cancer through induction of calreticulin exposure by Tras-Permut CrossMab. Oncoimmunology (2015) 4(3):e994391. 10.4161/2162402x.2014.994391 25949918 PMC4404837

[18] Li JY Perry SR Muniz-Medina V Wang X Wetzel LK Rebelatto MC. A biparatopic HER2-targeting antibody-drug conjugate induces tumor regression in primary models refractory to or ineligible for HER2-targeted therapy. Cancer Cell (2016) 29(1):117–29. 10.1016/j.ccell.2015.12.008 26766593

[19] Zhang Y Wang L Chong X Yu X Meng Y Dong J. A bispecific anti-ErbB2 antibody potently induces ErbB2 internalization and suppresses ErbB2-overexpressing tumor growth. Biochem Biophys Res Commun (2016) 477(4):755–60. 10.1016/j.bbrc.2016.06.131 27363335

[20] Liu J Wu X Lin L Pan H Wang Y Li Y. Bp-Bs, a novel T-cell engaging bispecific antibody with biparatopic Her2 binding, has potent anti-tumor activities. Mol Ther Oncolytics (2019) 14:66–73. 10.1016/j.omto.2019.03.009 31020038 PMC6475711

[21] McDonagh CF Huhalov A Harms BD Adams S Paragas V Oyama S. Antitumor activity of a novel bispecific antibody that targets the ErbB2/ErbB3 oncogenic unit and inhibits heregulin-induced activation of ErbB3. Mol Cancer Ther (2012) 11(3):582–94. 10.1158/1535-7163.MCT-11-0820 22248472

[22] Malm M Bass T Gudmundsdotter L Lord M Frejd FY Stahl S. Engineering of a bispecific affibody molecule towards HER2 and HER3 by addition of an albumin-binding domain allows for affinity purification and in vivo half-life extension. Biotechnol J (2014) 9(9):1215–22. 10.1002/biot.201400009 24678002

[23] Diermeier-Daucher S Ortmann O Buchholz S Brockhoff G. Trifunctional antibody ertumaxomab: Non-immunological effects on Her2 receptor activity and downstream signaling. MAbs (2012) 4(5):614–22. 10.4161/mabs.21003 PMC3499302 22820509

[24] Vaishampayan U Thakur A Rathore R Kouttab N Lum LG. Phase I study of anti-CD3 x Anti-Her2 bispecific antibody in metastatic castrate resistant prostate cancer patients. Prostate Cancer (2015) 2015:e285193. 10.1155/2015/285193 PMC4352947 25802762

[25] Kiewe P Hasmüller S Kahlert S Heinrigs M Rack B Marmé A. Phase I trial of the trifunctional anti-HER2× anti-CD3 antibody ertumaxomab in metastatic breast cancer. Clin Cancer Res (2016) 12(10):3085–91. 10.1186/s12885-016-2449-0 16707606

[26] Yu S Zhang J Yan Y Yao X Fang L Xiong H. A novel asymmetrical anti-HER2/CD3 bispecific antibody exhibits potent cytotoxicity for HER2-positive tumor cells. J Exp Clin Cancer Res (2019) 38(1):e355. 10.1186/s13046-019-1354-1 PMC6694677 31412896

[27] Mittal D Vijayan D Neijssen J Kreijtz J Habraken MMJM Van Eenennaam H. Blockade of ErbB2 and PD-L1 using a bispecific antibody to improve targeted anti-ErbB2 therapy. Oncoimmunology (2019) 8(11):e1648171. 10.1080/2162402X.2019.1648171 31646095 PMC6791437

[28] Hinner MJ Aiba RSB Jaquin TJ Berger S Dürr MC Schlosser C. Tumor-localized costimulatory T-cell engagement by the 4-1BB/HER2 bispecific antibody-anticalin fusion PRS-343. Clin Cancer Res (2019) 25(19):e5878. 10.1158/1078-0432.CCR-18-3654 31138587

[29] de Goeij BE Vink T Ten Napel H Breij EC Satijn D Wubbolts R. Efficient payload delivery by a bispecific antibody-drug conjugate targeting HER2 asnd CD63. Mol Cancer Ther (2016) 15(11):2688–97. 10.1158/1535-7163.mct-16-0364 27559142

[30] Wu X Demarest SJ. Building blocks for bispecific and trispecific antibodies. Methods (San Diego Calif). (2019) 154:3–9. 10.1016/j.ymeth.2018.08.010 30172007

[31] Wu C Ying H Grinnell C Bryant S Miller R Clabbers A. Simultaneous targeting of multiple disease mediators by a dual-variable-domain immunoglobulin. Nat Biotechnol (2007) 25(11):1290–7. 10.1038/nbt1345 17934452

[32] Gu J Yang J Chang Q Liu Z Ghayur T Gu J. Identification of anti-EGFR and anti-ErbB3 dual variable domains immunoglobulin (DVD-Ig) proteins with unique activities. PLoS One (2015) 10(5):e0124135. 10.1371/journal.pone.0124135 25997020 PMC4440733

[33] Piccione EC Juarez S Liu J Tseng S Ryan CE Narayanan C. A bispecific antibody targeting CD47 and CD20 selectively binds and eliminates dual antigen expressing lymphoma cells. MAbs (2015) 7(5):946–56. 10.1080/19420862.2015.1062192 PMC4623422 26083076

[34] Zeng J Liu R Wang J Fang Y. A bispecific antibody directly induces lymphoma cell death by simultaneously targeting CD20 and HLA-DR. J Cancer Res Clin Oncol (2015) 141(11):1899–907. 10.1007/s00432-015-1949-7 PMC11823833 25773122

[35] Khatri A Goss S Jiang P Mansikka H Othman AA. Pharmacokinetics of ABT-122, a TNF-α- and IL-17A-targeted dual-variable domain immunoglobulin, in healthy subjects and patients with rheumatoid arthritis: results from three phase I trials. Clin Pharmacokinetics. (2018) 57:613–23. 10.1007/s40262-017-0580-y 28744796

[36] Kosloski MP Goss S Wang SX Liu J Loebbert R Medema JK. Pharmacokinetics and tolerability of a dual variable domain immunoglobulin ABT-981 against IL-1alpha and IL-1beta in healthy subjects and patients with osteoarthritis of the knee. J Clin Pharmacol (2016) 56(12):1582–90. 10.1002/jcph.764 27150261

[37] Ding K Eaton L Bowley D Rieser M Chang Q Harris MC. Generation and characterization of ABBV642, a dual variable domain immunoglobulin molecule (DVD-Ig) that potently neutralizes VEGF and PDGF-BB and is designed for the treatment of exudative age-related macular degeneration. MAbs (2017) 9(2):269–84. 10.1080/19420862.2016.1268305 PMC5297536 27929753

[38] Craig RB Summa CM Corti M Pincus SH. Anti-HIV double variable domain immunoglobulins binding both gp41 and gp120 for targeted delivery of immunoconjugates. PLoS One (2012) 7(10):e46778. 10.1371/journal.pone.0046778 23056448 PMC3464217

[39] McGaraughty S Davis-Taber RA Zhu CZ Cole TB Nikkel AL Chhaya M. Targeting anti-TGF-beta therapy to fibrotic kidneys with a dual specificity antibody approach. J Am Soc Nephrol JASN (2017) 26(12):3616–26. 10.1681/asn.2017010013 PMC5698069 28827403

[40] Karaoglu Hanzatian D Schwartz A Gizatullin F Erickson J Deng K Villanueva R. Brain uptake of multivalent and multi-specific DVD-Ig proteins after systemic administration. MAbs (2018) 10(5):765–77. 10.1080/19420862.2018.1465159 PMC6150631 29771629

[41] Li Y Hickson JA Ambrosi DJ Haasch DL Foster-Duke KD Eaton LJ. ABT-165, a dual variable domain immunoglobulin (DVD-Ig) targeting DLL4 and VEGF, demonstrates superior efficacy and favorable safety profiles in preclinical models. Mol Cancer Ther (2018) 17(5):1039–50. 10.1158/1535-7163.Mct-17-0800 29592882

[42] Ibrahim S Girault A Ohresser M Lereclus E Paintaud G Lecomte T. Monoclonal antibodies targeting the IL-17/IL-17RA axis: an opportunity to improve the efficiency of anti-VEGF therapy in fighting metastatic colorectal cancer. Clin Colorectal Cancer (2018) 17(1):109–13. 10.1016/j.clcc.2017.10.003 29153431

[43] Fleischmann RM Bliddal H Blanco FJ Schnitzer TJ Peterfy C Chen S. A phase II trial of lutikizumab, an anti-interleukin-1α/β dual variable domain immunoglobulin, in knee osteoarthritis patients with synovitis. Arthritis Rheumatol (Hoboken NJ). (2019) 71(7):1056–69. 10.1002/art.40840 30653843

[44] Chengbin Wu HY Bose S Miller R Medina L Ghayur LST. Molecular construction and optimization of anti-human IL-1α/β dual variable domain immunoglobulin (DVD-IgTM) molecules. mAbs (2009) 1(4):339–47. 10.4161/mabs.1.4.8755 PMC2726605 20068402

[45] DiGiammarino EL Harlan JE Walter KA Ladror US Edalji RP Hutchins CW. Ligand association rates to the inner-variable-domain of a dual-variable-domain immunoglobulin are significantly impacted by linker design. mAbs (2011) 3(5):487–94. 10.4161/mabs.3.5.16326 PMC3225853 21814039

[46] Hosseini Ghatar R Soltantoyeh T Bahadori T Golara M Hassannia H Khosravi Eghbal R. Epitope mapping of human HER2 specific mouse monoclonal antibodies using recombinant extracellular subdomains. Asian Pacific J Cancer Prev APJCP (2017) 18(11):3103–10. 10.22034/apjcp.2017.18.11.3103 PMC5773798 29172286

[47] Golsaz-Shirazi F Amiri MM Farid S Bahadori M Bohne F Altstetter S. Construction of a hepatitis B virus neutralizing chimeric monoclonal antibody recognizing escape mutants of the viral surface antigen (HBsAg). Antiviral Res (2017) 144:153–63. 10.1016/j.antiviral.2017.06.013 28641998

[48] Soltantoyeh T Bahadori T Hosseini-Ghatar R Khoshnoodi J Roohi A Mobini M. Differential effects of inhibitory and stimulatory anti-HER2 monoclonal antibodies on AKT/ERK signaling pathways. Asian Pacific J Cancer Prev APJCP (2018) 19(8):2255–62. 10.22034/apjcp.2018.19.8.2255 PMC6171393 30139234

[49] Scheuer W Friess T Burtscher H Bossenmaier B Endl J Hasmann M. Strongly enhanced antitumor activity of Trastuzumab and Pertuzumab combination treatment on HER2-positive human xenograft tumor models. Cancer Res (2009) 69(24):9330–6. 10.1158/0008-5472.CAN-08-4597 19934333

[50] Nahta R Hung M-C Esteva FJ. The HER-2-targeting antibodies trastuzumab and pertuzumab synergistically inhibit the survival of breast cancer cells. Cancer Res (2004) 64(7):2343–46. 10.1158/0008-5472.CAN-03-3856 15059883

[51] Jakob CG Edalji R Judge RA DiGiammarino E Li Y Gu J. Structure reveals function of the dual variable domain immunoglobulin (DVD-Ig) molecule. MAbs (2013) 5(3):358–63. 10.4161/mabs.23977 PMC4169029 23549062

[52] Lacy SE Wu C Ambrosi DJ Hsieh C-M Bose S Miller R. Generation and characterization of ABT-981, a dual variable domain immunoglobulin (DVD-Ig(TM)) molecule that specifically and potently neutralizes both IL-1α and IL-1β. mAbs (2015) 7(3):605–19. 10.1080/19420862.2015.1026501 PMC4622731 25764208

[53] Gu J Ghayur T. Generation of dual-variable-domain immunoglobulin molecules for dual-specific targeting. Methods Enzymol (2012) 502:25–41. 10.1016/B978-0-12-416039-2.00002-1 22208980

[54] Tang Y Lou J Alpaugh RK Robinson MK Marks JD Weiner LM. Regulation of Antibody-Dependent Cellular Cytotoxicity by IgG Intrinsic and Apparent Affinity for Target Antigen. J Immunol (2007) 179(5):2815. 10.4049/jimmunol.179.5.2815 17709495

[55] Velders MP van Rhijn CM Oskam E Fleuren GJ Warnaar SO Litvinov SV. The impact of antigen density and antibody affinity on antibody-dependent cellular cytotoxicity: relevance for immunotherapy of carcinomas. Br J Cancer (1998) 78(4):478–83. 10.1038/bjc.1998.518 PMC2063081 9716030

[56] Sakahara H Endo K Koizumi M Nakashima T Kunimatsu M Watanabe Y. Relationship between in vitro binding activity and in vivo tumor accumulation of radiolabeled monoclonal antibodies. J Nucl Med (1988) 29(2):235–40. 10.1000/res#test 3162255

