# Supplementary material for: A Novel Anti-HER2 Bispecific Antibody With Potent Tumor Inhibitory Effects In Vitro and In Vivo
Source: Front Immunol. 2021 Feb 17;11:600883. doi: 10.3389/fimmu.2020.600883 (PMC7927792; doi:10.3389/fimmu.2020.600883)
Supplement: Supplementary file 1 [file DataSheet_1.pdf]

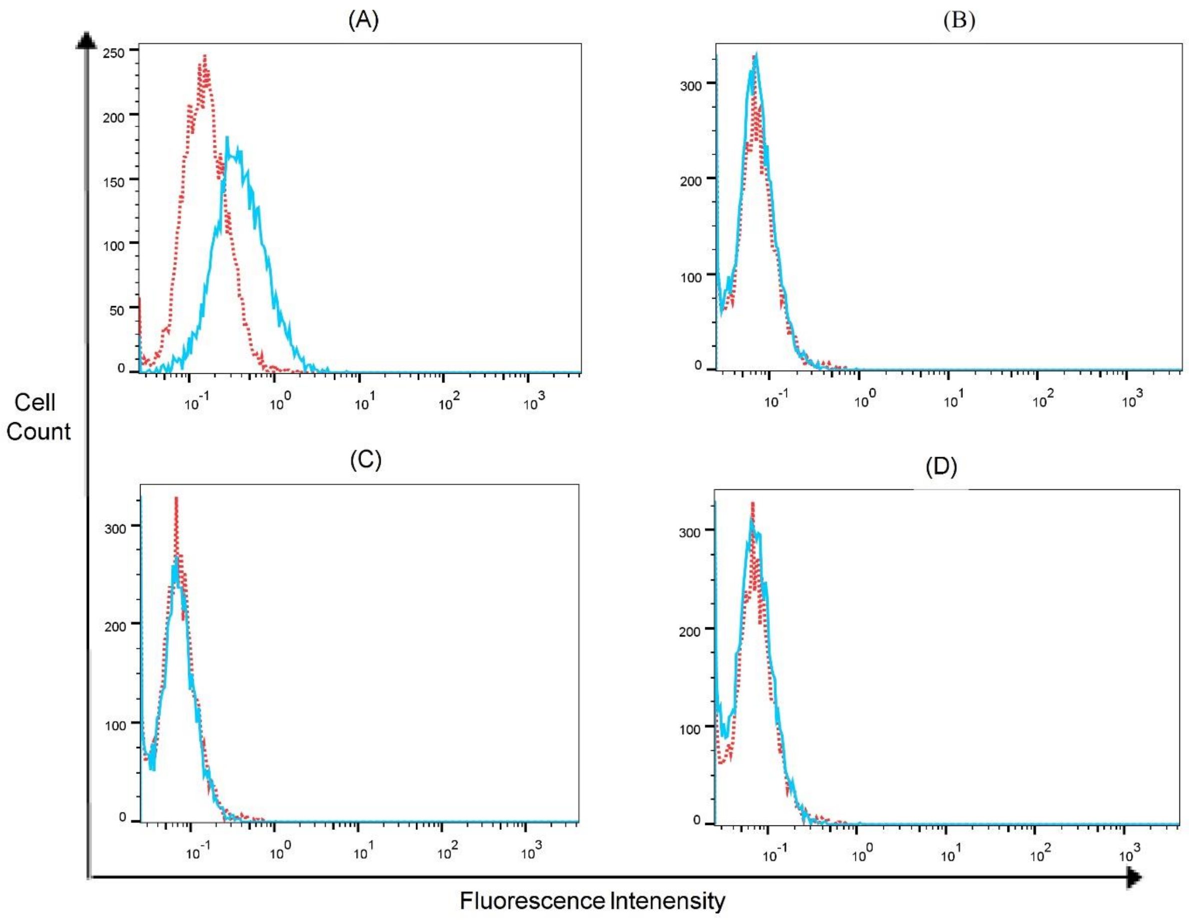

Supplementary Figure 1. Binding of BiHT and their parental antibodies to mouse HER2-expressing cell line. 3T3-L1 cells were treated with rabbit anti-HER2 polyclonal antibody (A), BiHT (B), trastuzumab (C) or hersintuzumab (D), as primary antibodies. Then FITC labeled anti-rabbit Ig polyclonal antibody (A) or FITC labeled anti-human Ig polyclonal antibody (B-D) were used as the secondary antibody. The final results were analyzed by flow cytometry. Normal rabbit IgG (A) and chimeric anti-hepatitis B mAb harboring human IgG1/κ (B-D) were employed as negative controls (dotted lines).
