# Supplementary material for: A Novel Anti-HER2 Bispecific Antibody With Potent Tumor Inhibitory Effects In Vitro and In Vivo
Source: Front Immunol. 2021 Feb 17;11:600883. doi: 10.3389/fimmu.2020.600883 (PMC7927792; doi:10.3389/fimmu.2020.600883)
Supplement: Supplementary file 2 [file DataSheet_2.pdf]

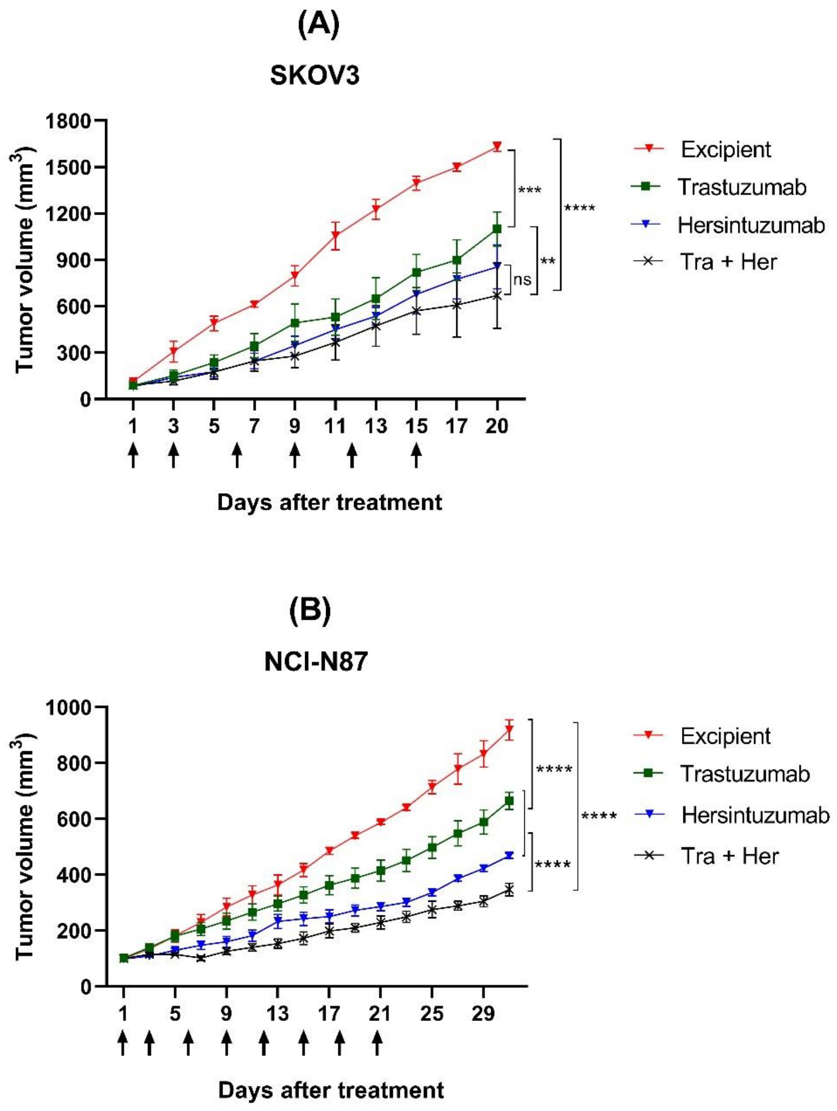

Supplementary Figure 2. Comparative anti-proliferative effects of trastuzumab, hersintuzumab and their combination in nude mice. SKOV-3 (A) and NCI-N87 (B) xenograft tumor bearing nude mice were treated twice a week with a combination of trastuzumab plus hersintuzumab (5 mg/kg of each), or each mAb alone (10 mg/kg) for 2-3 consecutive weeks. The control group received excipient alone. Tumor sizes were measured three times a week. Data is shown as mean ± SEM. ****P< 0.0001, ***P< 0.001, **P< 0.01, ns: non-significant. Tra: trastuzumab. Her: hersintuzumab. Arrows show timing of mAb administration.
