# Supplementary material for: A Novel Anti-HER2 Bispecific Antibody With Potent Tumor Inhibitory Effects In Vitro and In Vivo
Source: Front Immunol. 2021 Feb 17;11:600883. doi: 10.3389/fimmu.2020.600883 (PMC7927792; doi:10.3389/fimmu.2020.600883)
Supplement: Supplementary file 3 [file Table_1.docx]

**Supplementary Table 1)** Statistical significance of the anti-proliferative effects of the BsAbs and their parental mAbs on different cell lines. ns: not significant

| Tukey's multiple comparisons test | Mean Diff. | Summary | Adjusted p value |
| --- | --- | --- | --- |
| **BT-474 cell line** |  |  |  |
| Trastuzumab:133 nM vs. Combination:133 nM | -34.28 | **** | <0.0001 |
| Trastuzumab:1.33 nM vs. Combination:1.33 nM | -26.65 | **** | <0.0001 |
| Trastuzumab:0.27 nM vs. Combination:0.27 nM | -26.56 | **** | <0.0001 |
| Hersintuzumab:133 nM vs. Combination:133 nM | -55.65 | **** | <0.0001 |
| Combination:133 nM vs. BiHT:133 nM | 24.65 | **** | <0.0001 |
| Combination:6.67 nM vs. BiHT:6.67 nM | 28.37 | **** | <0.0001 |
| Combination:1.33 nM vs. BiHT:1.33 nM | 3.979 | ns | 0.9929 |
| Combination:0.27 nM vs. BiHT:0.27 nM | 5.522 | ns | 0.9671 |
| BiHT:1.33 nM vs. BiTH:1.33 nM | 29.08 | **** | <0.0001 |
| BiHT:0.27 nM vs. BiTH:0.27 nM | 31.03 | **** | <0.0001 |
|  | | | |
| **SKOV-3 cell line** |  |  |  |
| Trastuzumab:6.67 nM vs. Hersintuzumab:6.67 nM | 11.96 | * | 0.0387 |
| Combination:6.67 nM vs. BiHT:6.67 nM | 4.979 | ns | 0.7413 |
| Combination:1.33 nM vs. BiHT:133 nM | -22.27 | **** | <0.0001 |
| Combination:1.33 nM vs. BiHT:0.27 nM | 14.23 | ** | 0.0032 |
| BiHT:6.67 nM vs. BiTH:6.67 nM | 10.57 | * | 0.0166 |
| BiHT:1.33 nM vs. BiTH:1.33 nM | 26.19 | **** | <0.0001 |
| BiHT:0.27 nM vs. BiTH:0.27 nM | 10.07 | * | 0.0102 |
|  | | | |
| **NCI-N87 cell line** |  |  |  |
| Trastuzumab:133 nM vs. Combination:133 nM | -21.50 | ** | 0.0033 |
| Combination:133 nM vs. BiHT:133 nM | 15.50 | ns | 0.1464 |
| Combination:0.27 nM vs. BiHT:0.27 nM | -19.25 | ** | 0.0045 |
| BiHT:0.27 nM vs. BiTH:0.27 nM | 28.89 | **** | <0.0001 |
|  | | | |
| **HCC-1954 cell line** |  |  |  |
| Trastuzumab:1.33 nM vs. BiHT:1.33 nM | -19.13 | ** | 0.0057 |
| Combination:1.33 nM vs. BiHT:1.33 nM | -16.68 | *** | 0.0002 |
| BiHT:6.67 nM vs. BiTH:6.67 nM | 16.79 | * | 0.0121 |
| BiHT:1.33 nM vs. BiTH:1.33 nM | 19.55 | **** | <0.0001 |
